# Supplementary material for: The interactome of intact mitochondria by cross-linking mass spectrometry provides evidence for coexisting respiratory supercomplexes
Source: Mol Cell Proteomics. 2017 Dec 8;17(2):216–32. doi: 10.1074/mcp.RA117.000470 (PMC5795388; doi:10.1074/mcp.RA117.000470)
Supplement: Supplemental Data [file supp_RA117.000470_133922_0_supp_23978_fzffwf.zip › spectra_annotation/mito_spectra_annotation/25-1-3-1-3-1.pdf]

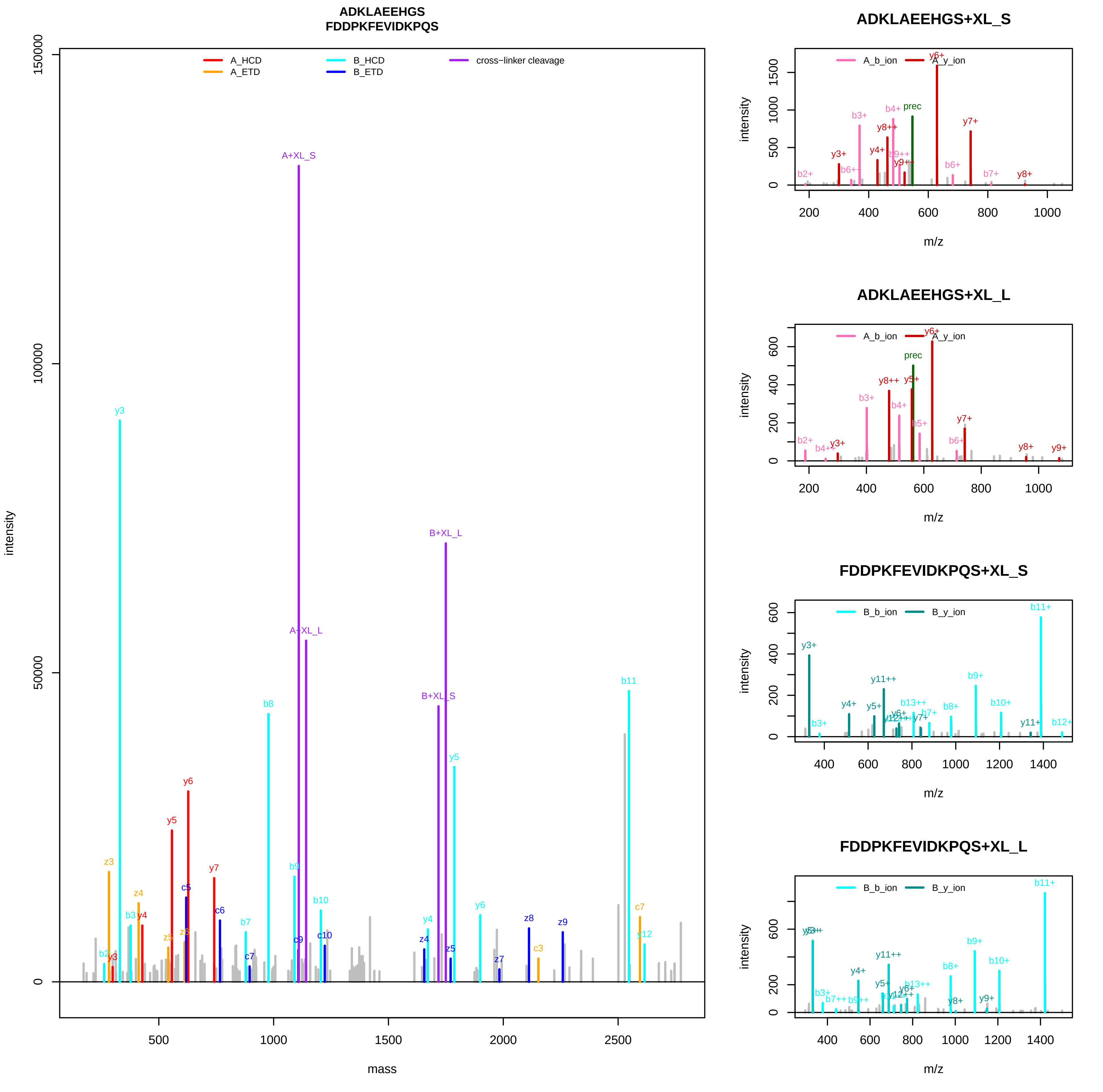

ADKLAEEHGS
FDDPKFEVIDKPQS
A_HCD
A_ETD
B_HCD
B_ETD
cross−linker cleavage
intensity
mass
0
50000
100000
150000
500
1000
1500
2000
2500
A+XL_S
A+XL_L
B+XL_L
B+XL_S
y3
b2
b3
z3
z4
y4
z5
z6
y5
y6
c5
c6
y7
b7
c7
b8
b9
c9
b10
c10
y4
z4
y5
z5
y6
z7
z8
c3
z9
b11
c7
y12
ADKLAEEHGS+XL_S
A_b_ion
A_y_ion
intensity
m/z
prec
ADKLAEEHGS+XL_L
FDDPKFEVIDKPQS+XL_S
B_b_ion
B_y_ion
FDDPKFEVIDKPQS+XL_L
